# Mice on a high-fat diet have reduced immunopathology and an altered immune response during respiratory syncytial virus infection

**DOI:** 10.1128/mbio.00689-26

**Published:** 2026-04-30

**Authors:** Kendall T. Whitt, Dorothea R. Morris, Pamela H. Brigleb, Lauren Rowland, Heather Sheppard, Stacey Schultz-Cherry

**Affiliations:** 1 Department of Host-Microbe Interactions, St. Jude Children’s Research Hospital 5417, Memphis, Tennessee, USA; 2 St. Jude Graduate School of Biomedical Sciences, Memphis, Tennessee, USA; Duke University School of Medicine, Durham, North Carolina, USA

**Keywords:** respiratory syncytial virus, obesity, disease severity, immune response

## Abstract

Obesity has been identified as an independent risk factor for increased morbidity and mortality during pandemic H1N1 influenza and SARS-CoV-2 infections. Various studies have determined that obese hosts have dysregulated antiviral responses driven by systemic low-grade inflammation, resulting in prolonged viral replication, induction of proinflammatory cytokines during the disease resolution phase, and profound lung parenchymal damage. However, the impact of obesity on disease outcomes in the context of other viral respiratory infections remains unclear. In this study, we sought to understand how obesity affects disease progression during respiratory syncytial virus (RSV) infection using an obese mouse model in which male C57BL/6 mice were fed a high-fat diet (HFD). Mice fed a standard chow diet served as controls. We found that HFD mice exhibited reduced weight loss, illness scores, and histopathological changes after intranasal inoculation with RSV A2. Investigation of potential mechanisms underlying this reduction in severity revealed reduced viral load in obese mice, along with altered innate and adaptive immune responses. Specifically, HFD mice had reduced proinflammatory cytokines in the lungs early in infection and increased CD4^+^ T cells late in infection. HFD mice also had reduced M2 macrophages in the lungs at 7 days post-infection. Notably, HFD mice infected with RSV did not exhibit aberrant proinflammatory cytokine secretion late in infection, as seen with influenza, likely due to effective viral control. In conclusion, HFD mice exhibited reduced disease severity during RSV infection, associated with decreased viral load and an attenuated but sufficient antiviral response.

**IMPORTANCE:**

Obesity has been shown to induce dysregulated antiviral responses during influenza infections, resulting in extensive morbidity and mortality. No studies to date have investigated how obesity-induced immune dysregulation affects respiratory syncytial virus (RSV) disease progression. RSV has a high global burden, inflicting millions of infections and tens of thousands of deaths yearly, most notably among the very young, the elderly, and those with comorbidities. It is essential to understand how risk factors, such as obesity, affect disease progression to ensure appropriate protection and care for patients. Here, we demonstrate that male C57BL/6 mice fed a high-fat diet had lower viral loads and attenuated inflammatory responses during RSV infection, resulting in reduced morbidity and immunopathology. This pilot study advances our understanding of how obesity affects pulmonary antiviral immunity to RSV and, concurrently, further elucidates RSV pathogenesis.

## INTRODUCTION

Obesity is a global burden, with rates tripling since 1975 and over 4 million deaths per year attributed to obesity-related complications (1). While the link between obesity and conditions such as cardiovascular disease or diabetes is well established (2), there is increasing evidence that obesity also negatively affects the response to respiratory infections (3, 4). Outcomes from the 2009 influenza H1N1 and the 2019 SARS-CoV-2 pandemics identified obesity as an independent risk factor for increased probability of hospitalization, mechanical ventilation, and mortality following infection (5, 6).

This correlation between obesity and poor response to viral respiratory infections has been linked to immune modulation in obese hosts (7). Adipokine production by excess adipose tissues results in higher baseline systemic inflammation, thereby contributing to dysregulated antiviral responses and impaired wound healing in the lungs upon infection (8–10). Studies from our laboratory and others comparing high-fat diet (HFD) obese mice to lean wild-type mice have found that during influenza A virus (IAV) infection, obese mice exhibit blunted type I interferon (IFN) production, delayed secretion of proinflammatory cytokines at the site of infection, decreased production of anti-inflammatory cytokines, and impaired recruitment of immune cells (9, 11, 12). This dysregulated antiviral response leads to increased lung inflammation later in infection and prolonged viral shedding (10, 13), ultimately causing severe immunopathology and lung damage, increased susceptibility to secondary bacterial infections, and death (11, 14).

Although numerous studies have examined the effects of obesity on IAV infection, the role of obesity on the pathogenesis of other respiratory viruses remains understudied. For example, the impact of obesity on the progression and outcomes of human respiratory syncytial virus (RSV) infection is poorly understood, and conclusions from clinical studies are often conflicting, with some groups finding no correlation between obesity and RSV disease progression (15, 16). RSV is a leading cause of hospitalization for lower respiratory tract infections (LRTIs) in infants, the elderly, and the immunocompromised (17, 18), with an exaggerated and aberrant immune response being attributed as the primary driver of disease severity (19). Individuals with obesity are among the recommended populations for RSV vaccination (20). However, there are relatively few clinical reports that consider obesity among potential drivers of disease severity, and no reports evaluating RSV pathogenesis in obese animal models have been published to our knowledge. As adult and pediatric obese populations continue to expand worldwide, it is essential to understand how dysregulated immunity in obesity influences RSV disease progression.

Given the identification of dysregulated antiviral immunity in obese hosts during IAV infection and conflicting reports on the influence of obesity on RSV disease progression, we sought to determine how obesity affects RSV pathogenesis in a HFD mouse model. Importantly, because RSV disease manifestations are driven by host-mediated immunopathology rather than virus-mediated damage, as seen in IAV infections, the influence of dysregulated immunity in obesity during RSV infection poses an interesting question (21–23). Here, we show that obese mice infected with RSV A2 exhibited reduced disease severity, decreased viral loads, and a reduced inflammatory pulmonary microenvironment compared to lean controls. This novel study evaluates RSV disease progression in HFD mice and further elucidates the nuances of antiviral immunity in obese hosts that warrant further exploration.

## MATERIALS AND METHODS

### RSV propagation and titration

RSV A2 was acquired from ATCC (American Type Culture Collection, VR-1540P) and inoculated at a multiplicity of infection (MOI) of 0.05 in HEp2 cells at 80% confluency to produce virus stocks. The virus was propagated for 42 h until extensive syncytium formation was observed. The supernatant and cells were collected and centrifuged at 2,900 rpm at 4°C for 15 min, and the supernatant was concentrated using Amicon Ultra Centrifugal Filters, 100 kDa MWCO (Millipore Sigma, UFC910024). The virus stock was aliquoted, snap-frozen, and stored at −80°C until needed. Infectious viral titers for virus stocks and infected mouse lungs were determined by methylcellulose plaque assay as previously described (24).

### Mouse husbandry and infection

Male C57BL/6 mice were purchased from Jackson Laboratory at 4–6 weeks of age and randomly divided into standard chow diet (10 kcal% fat, Research Diets Inc, D12450B) and high-fat chow diet (60 kcal% fat, Research Diets Inc, D12492) groups. Mice were provided with food and water *ad libitum* and monitored for 14–16 weeks until those on the high-fat diet developed serological markers of metabolic dysfunction, as previously described (25).

To identify an infectious dose of RSV A2 that induced a maximum weight loss of 10% or more of original body weight, 20-week-old male C57BL/6 mice fed a standard diet were anesthetized with 90 mg/kg of Ketamine and 7.5 mg/kg of Xylazine administered by intraperitoneal injection and intranasally inoculated with 50 µL of 5 × 10^6^ or 10^7^ plaque-forming units (PFUs) diluted in PBS. Weights were recorded daily for 14 days. An inoculum of 10^7^ PFUs was determined to induce ideal illness in male C57BL/6 mice.

Twenty to 22-week-old male mice that were on a standard chow diet (SD) or a high-fat chow diet (HFD) for 14–16 weeks were anesthetized with 90 mg/kg of Ketamine and 7.5 mg/kg of Xylazine administered by intraperitoneal injection. Mice were then intranasally inoculated with 50 µL of 10^7^ PFUs of RSV A2 diluted in PBS. Mock-infected mice were intranasally inoculated with 50 µL of PBS. The mice were weighed daily and scored for illness. Illness scores were visually determined using a standardized 0–5 scoring system (0 = no disease, 1 = light ruffling of fur, 2 = heavy ruffling of fur, 3 = ruffled fur and hunched posture, 4 = ruffled fur, hunched posture, and inactivity, 5 = death) (26).

Lungs were collected on 1, 4, 7, and 14 days post-infection (dpi) to determine viral titers and cytokine quantification. Mock- or virus-infected mice were euthanized with carbon dioxide in accordance with ethical guidelines at the indicated time points. Bronchoalveolar lavage fluid (BALF) was collected, and the whole lung was harvested before being separated into left and right lobes. The left lobe was placed in formalin for at least 24 h before being processed and paraffin-embedded by the SJCRH Comparative Pathology Core, detailed below. The right lobe was snap-frozen and later homogenized in PBS to determine viral titers and to quantify cytokines.

### Whole-body plethysmography

Airway function of mice was recorded daily using a non-invasive whole-body plethysmograph (Data Sciences International, New Brighton, MN, USA). Unrestrained, conscious mice were placed in individual chambers, and enhanced pause (Penh) was recorded and averaged over a 5-min period to determine airway obstruction (27, 28). The measurements were acquired prior to infection and daily for 14 dpi. Because the mice were not challenged with methacholine, pre-infected measurements were used for baseline values.

### Histopathology

Lungs were fixed in 10% neutral buffered formalin, embedded in paraffin, and sectioned at 4 μm. Sections were mounted on positively charged glass slides (Superfrost Plus; Thermo Fisher Scientific, 12-550-15) and dried at 60°C for 20 min. Slides were then deparaffinized and stained with hematoxylin and eosin (Richard-Allan Scientific, HE). H&E staining and cover slipping were performed using the HistoCore SPECTRA Workstation (Leica Biosystems). Serial unstained sections were used for single-plex immunohistochemistry (IHC). Immunolabeling with the anti-RSV nucleocapsid (N) antibody (ViroStat #0601, Goat Polyclonal; primary antibody diluted 1:5,000) was performed on the Ventana Discovery Ultra autostainer (Roche, Indianapolis, IN, USA). Heat-induced epitope retrieval was carried out using Cell Conditioning 1 (CC1, Cat. No. 950-224) for 32 min at 37°C. Visualization was achieved using a biotinylated Rabbit Anti-Goat IgG (H+L) secondary antibody (Vector Laboratories, BA5000), followed by DISCOVERY OmniMap anti-rabbit HRP (760-4311) and the DISCOVERY ChromoMap DAB kit (760-159). Slides were scanned at 20× and 32× magnification using a Pannoramic 250 Flash III scanner (3DHistech) and analyzed with HALO software v3.6.4134 (Indica Labs). The slides were evaluated and graded under light microscopy by a board-certified veterinary pathologist with expertise in mouse lung pathology, who was unaware of the diet groups or infection status of the animals. Histopathological scores were determined as follows: 0, no notable findings; 1, minimal; 2, mild; 3, moderate; and 4, marked.

### Albumin and cytokine measurements

BALF albumin concentration was determined using the mouse Albumin ELISA kit (Abcam, ab108792). Mouse cytokines were measured in lung homogenates using the LEGENDplex mouse antivirus response panel (Biolegend, 740622). The above assays were performed according to the manufacturer’s instructions. For the Albumin ELISA kit, samples from mock-infected mice were diluted 1:5,000, and samples from RSV-infected mice were diluted 1:20,000. For the LEGENDplex panel, undiluted samples were tested.

### Real-time quantitative PCR

RNA was extracted from mouse lung homogenates using the QIAamp viral RNA mini kit (Qiagen, 52906) according to the manufacturer’s protocol and stored at −80°C. RSV N gene was detected by real-time quantitative polymerase chain reaction (RT-qPCR) using TaqMan Fast Virus 1-Step Master Mix for qPCR (ThermoFisher Scientific, 4444434). Samples were run on the Bio-Rad C1000 Touch Thermal Cycler under the following conditions: 45°C for 10 min, 95°C for 10 min, and 45 cycles of 95°C for 15 s, and 55°C for 1 min. Use of the RSV N gene forward primer (CCCCACTTTATAGATGTTTTTGTTCA), reverse primer (TCCTGCAAAAATCCC-TTCAACT), and probe (FAM-TATAGCACA/ZEN/ATCTTCTACCAGAGG-TAMRA) was previously described (29). Samples were compared to standard dilutions of an RSV N gBlock to calculate the total gene copy numbers in each sample.

### Tissue and sample processing for flow cytometry

Whole lungs were harvested from mock- and RSV-infected mice at the indicated time points and immediately placed into 1.5 mL serum-free RPMI on ice. Lungs were finely minced with scissors and digested with 575 U/mL Collagenase IV (Worthington Biochemical, LS004189) and 60 U/mL DNase I (Worthington Biochemical, LS002007) in 1.5 mL of RPMI for 30 min at 37°C on a shaker. Samples were passed through a 100 µm strainer to generate a single-cell suspension and subjected to red blood cell lysis (Thermo Fisher, 00-4333-57).

Single-cell suspensions were counted, and 2 × 10^6^ cells were aliquoted into polypropylene round-bottom FACS tubes. Samples were first blocked with TruStain FcX PLUS (Biolegend, 156604) at 1:300 in FACS buffer (PBS, 5% FBS) for 10 min, and then stained with the viability dye Zombie NIR (Biolegend, 423106) at 1:800 in PBS without FBS for 15 min. Next, the samples were stained with the antibodies at the stated concentrations listed in Tables S1 or S2 for 45 min. All blocking and staining steps were performed on ice and protected from light. After staining, cells were fixed with BD Cytofix/Cytoperm kit (BD Sciences, 554714). Samples were run on the Cytek Aurora and analyzed using FlowJo software (Tree Star, Ashland, OR, USA).

### Intracellular cytokine staining

After counting the single-cell suspensions, 0.5 × 10^6^ cells were aliquoted in duplicate into a 24-well plate. The cells were stimulated with PMA and Ionomycin (Biolegend, 423301) in complete RPMI (RPMI, 5% FBS, Glutamax, Penicillin/Streptomycin) at 37°C. Brefeldin A (Biolegend, 420601) was added after 4 h, and the cells were incubated at 37°C for an additional 2 h. After a total of 6 h, cells were transferred to polypropylene round-bottom FACS tubes and washed twice with ice-cold FACS buffer. Surface staining was performed as detailed above, using the antibodies at the stated concentrations listed in Table S1. Cells were fixed with FOXp3/Transcription Factor staining buffer set (ThermoFisher Scientific, 00-5523-00) and subsequently stained with the antibodies listed in Table S1. Samples were run on Cytek Aurora and analyzed using FlowJo software.

### Statistical analysis

Data were organized in Microsoft Excel and plotted and analyzed in GraphPad Prism 10. Statistical significance was determined by an unpaired *t*-test or a mixed-effects model, followed by Tukey’s multiple comparison test. Data in the figures are displayed as means with standard deviation. Statistical significance is designated by an asterisk, with the following indications: *P < 0.05, **P < 0.01, ***P < 0.001, and ****P < 0.0001.

## RESULTS

### Disease severity is mitigated in RSV-infected HFD mice

**Fig 1:**
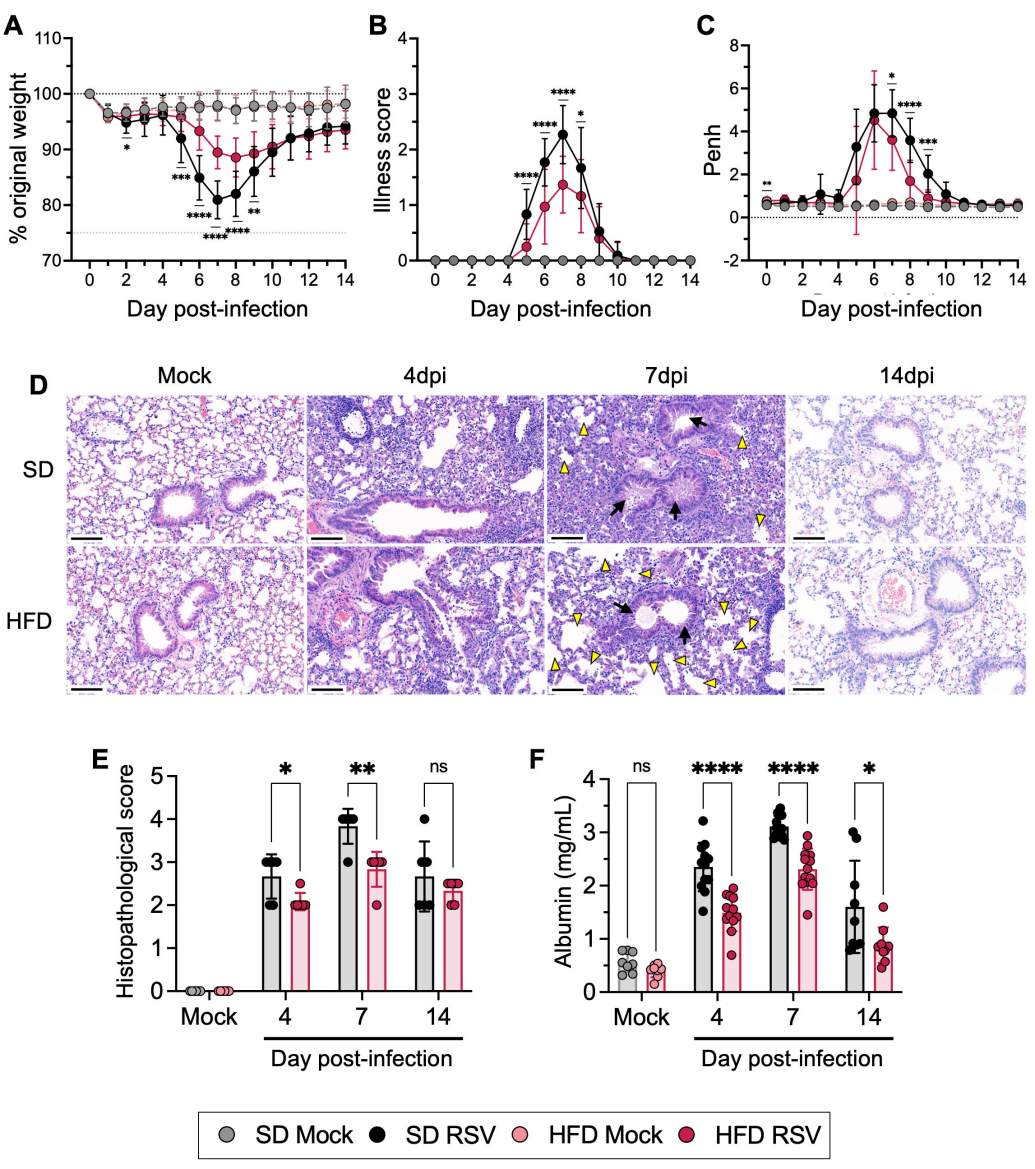
Mice on a high-fat diet have reduced weight loss and illness scores and improved histopathological changes during RSV infection. Mice fed a standard diet or a high-fat diet for a minimum of 14 weeks were infected intranasally with RSV A2 and monitored for 14 days. (**A**) Daily body weight measurements are plotted as a percentage of the original body mass. *N* = 5 mice/diet group/experiment, eight experiments. (**B**) Daily illness scores were recorded post-challenge. *N* = 5 mice/diet group/experiment, eight experiments. (**C**) Daily Penh was recorded post-challenge. *N* = 5 mice/diet group/experiment, four experiments. (**D**) Representative lung biopsies stained with hematoxylin and eosin. Images were captured at 15×, and the scale bar equals 100 µm. Yellow arrowheads denote patent alveoli at day 7 post-infection. Black arrows denote bronchioles at day 7 post-infection, with increased debris in the SD lung. (**E**) Histopathological scores were determined by a board-certified pathologist. *N* = 3 mice/diet group/experiment, two experiments. (**F**) Albumin concentration in bronchoalveolar lavage fluid determined by ELISA. *N* = 5 mice/diet group/experiment, three experiments. The results were plotted as mean ± SD. Statistical significance for each time point across all parameters was determined using a mixed-effects model with Tukey-Kramer multiple comparisons. Asterisks represent *P* values for SD RSV compared to HFD RSV; *P < 0.05, **P < 0.01, ***P < 0.001, ****P < 0.0001, ns = not significant.

In this study, we sought to understand how a HFD in mice affects the progression of RSV LRTI. We hypothesized that mice on a HFD diet would fare significantly worse than SD mice, as observed with IAV. Contrary to our hypothesis, HFD mice lost significantly less weight than SD mice on 5–9 dpi (Fig. 1A). In addition to losing a smaller percentage of weight, HFD mice also had significantly lower illness scores at 5–8 dpi, with the mice appearing more energetic, better groomed, and less hunched than SD mice (Fig. 1B). Finally, we observed that RSV infection had less impact on pulmonary function in HFD mice, as measured by whole-body plethysmography. HFD mice had significantly lower Penh (Fig. 1C) than SD mice during peak disease, indicating reduced airway obstruction (28).

To determine whether the reduced morbidity and airway obstruction measured in HFD mice was associated with decreased lung damage, we next evaluated lung pathology via histology (Fig. 1D and E; Fig. S1). Interstitial pneumonia was reported in both diet groups, although it was more advanced in SD mice than in HFD mice (Fig. 1E). SD mice progressed to marked pneumonia by 7 dpi, with prominent fluid and debris accumulation in the bronchioles (Fig. 1D, black arrows), as reflected by elevated Penh measurements at this time (Fig. 1C). In comparison, HFD mice were graded as having moderate pneumonia and had less cellular infiltration in their airways (Fig. 1D, yellow arrowheads). By 14 dpi, airways appeared clear of debris, and cellular infiltrates were reduced in both diet groups, indicating disease resolution in SD and HFD mice (Fig. 1D and E).

We further evaluated lung integrity during infection by measuring albumin levels in bronchoalveolar lavage fluid (BALF). Higher albumin concentration in BALF serves as a surrogate marker of increased alveolar-capillary permeability (30) and is a key metric elevated during acute lung injury (31). HFD mice had significantly less albumin measured in BALF at 4 and 7 dpi compared to SD mice, indicating improved maintenance of the lung parenchyma (Fig. 1F). Albumin levels decreased in the BALF collected from SD and HFD mice by 14 dpi, suggesting disease resolution in both diet groups. Taken together, these findings indicate that HFD mice exhibited reduced morbidity and histopathological changes during RSV infection, thereby experiencing attenuated RSV LRTI.

### RSV replication is reduced in obese mice

**Fig 2:**
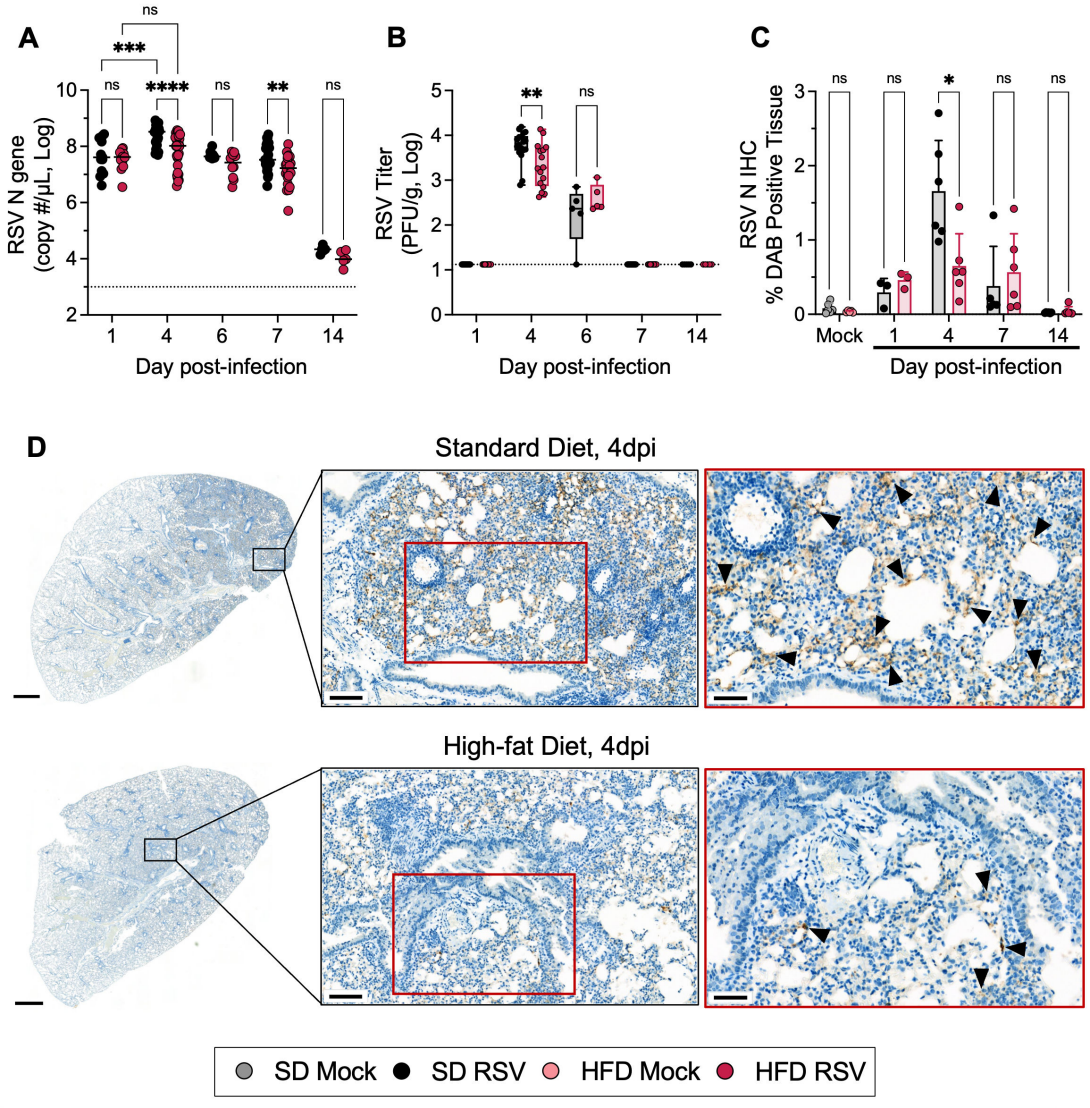
HFD mice have reduced viral replication and spread at peak infection. (**A**) Genome copies of RSV N gene were measured in lung homogenates. *N* = 5 mice/diet group/experiment, four experiments. (**B**) Replicating virus in lung homogenates was determined by plaque assay. *N* = 5 mice/diet group/experiment, four experiments. Negative results were entered as the limit of detection. (**C**) Quantification of RSV N protein IHC staining from histological slides. *N* = 3 mice/diet group/experiment, two experiments. (**D**) IHC staining for RSV N protein in representative lung biopsies. Images on the left were captured at 1×, and the scale bar equals 1 mm. Images in the middle were captured at 10×, and the scale bar equals 100 µm. Images on the right were captured at 20×, and the scale bar equals 50 µm. Positive RSV N staining is in brown, and the negative counterstain is in blue. Black arrows indicate focal RSV N staining near the alveolar interstitium. Statistical significance was determined using a mixed-effects model with Tukey-Kramer multiple comparisons. Asterisks represent *P* values for SD RSV compared to HFD RSV; *P < 0.05, **P < 0.01, ***P < 0.001, ****P < 0.0001, ns = not significant.

Although RSV is primarily a disease of host-mediated immunopathology rather than virus-induced cytopathology, the viral load during RSV infection influences the amplitude of the host immune response, thereby indirectly contributing to disease severity (21, 32, 33). Therefore, to begin elucidating potential mechanisms underlying the reduction in morbidity and histopathology in HFD mice, we evaluated the presence of RSV in the lungs of infected mice at 1, 4, 6, 7, and 14 dpi. We measured similar RSV N gene copy numbers in the lungs of HFD and SD mice at 1 dpi (Fig. 2A), indicating that both groups were adequately infected. At peak viral replication on 4 dpi, however, there was significantly less virus present in the lungs of HFD mice compared to SD mice, as determined through gene copy number (Fig. 2A), plaque titers (Fig. 2B), and IHC staining quantification (Fig. 2C). This reduction in viral presence was observed visually by representative histology slides of the left lung from SD and HFD mice collected at 4 dpi and stained for RSV N protein, with black arrowheads marking examples of positive RSV N staining (Fig. 2D). The lung from the SD mouse showed a diffuse staining pattern, indicating prominent replication and spread, while the lung from the HFD mouse had a sparse, multifocal staining pattern, supporting the findings of reduced replication in this diet group. At 6 dpi, genome copies and plaque titers began to decrease in both groups, and by 7 dpi, replicating titers were no longer detectable in either diet group (Fig. 2A and B). Mice in both diet groups had minimal viral genome copies present by 14 dpi, indicating similar viral clearance between the two groups (Fig. 2A). In conclusion, although RSV initially infected the lungs of mice in both diet groups to a similar extent, viral replication was reduced in HFD mice, resulting in decreased viral loads during peak viral replication.

### The lung microenvironment is less inflammatory in RSV-infected HFD mice

**Fig 3:**
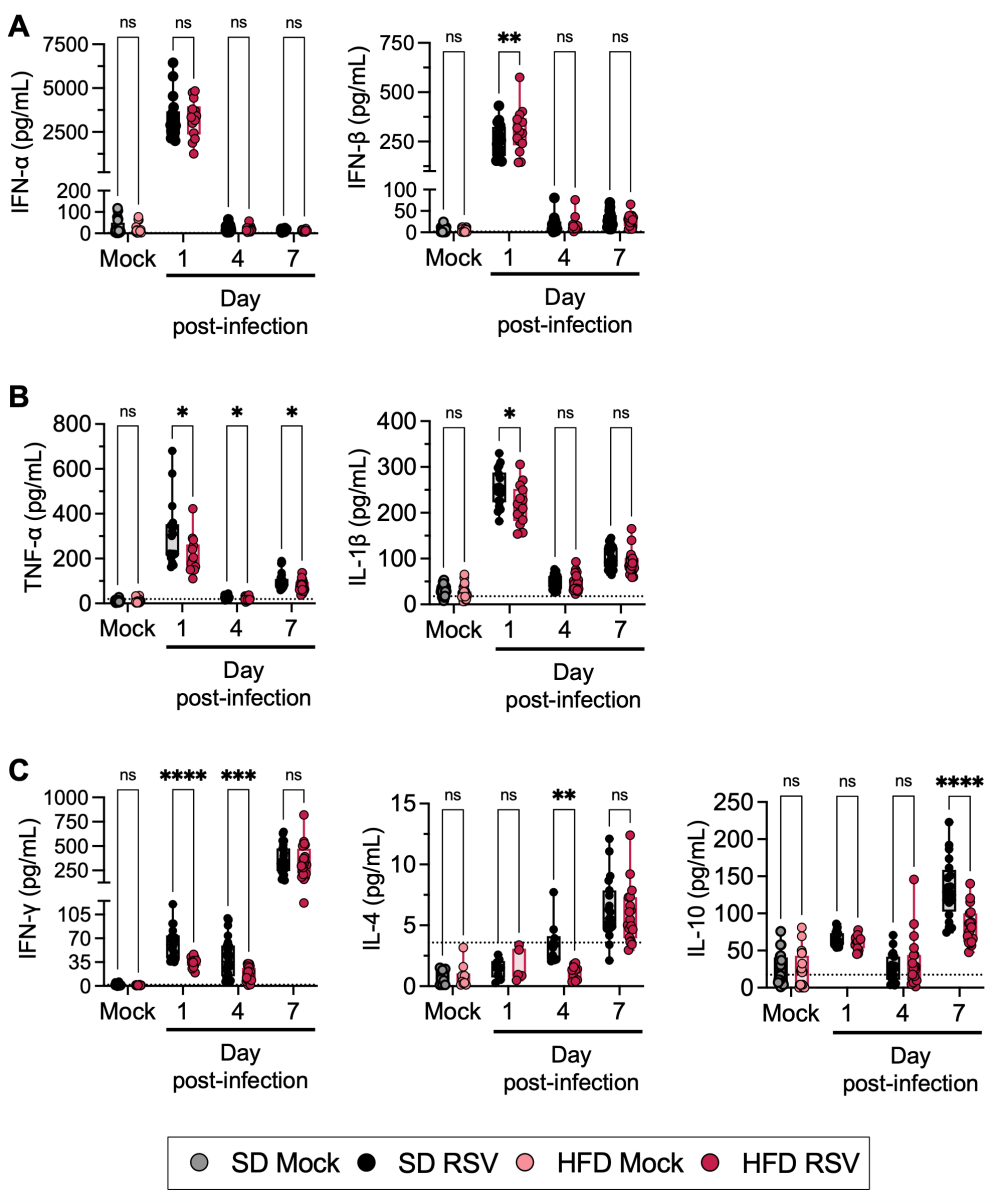
The immune microenvironment differs between RSV-infected SD and HFD mice. Cytokine concentrations measured in lung homogenates from mock- or RSV-infected mice fed a SD or HFD. (**A**) Type I interferons: IFN-α and IFN-β. (**B**) Proinflammatory cytokines: TNF-α and IL-1β. (**C**) T cell-associated cytokines: IFN-γ, IL-4, and IL-10. *N* = 5 mice/diet group/experiment, four experiments. Statistical significance for all parameters was determined using a mixed-effects model with Tukey-Kramer multiple comparisons. Asterisks represent *P* values for SD RSV compared to HFD RSV; *P < 0.05, **P < 0.01, ***P < 0.001, ****P < 0.0001, ns = not significant.

We observed differences in morbidity and histopathological changes between RSV-infected SD and HFD mice (Fig. 1), as well as reduced peak viral loads in the lungs of HFD mice (Fig. 2), leading us to hypothesize that HFD mice have an attenuated immune microenvironment in the lungs during this period. To evaluate antiviral and inflammatory responses in mice from both diet groups, we measured cytokine levels in lung homogenates from mock- and RSV-infected HFD and SD mice. Both diet groups had similar levels of type I IFNs in lung homogenates at 1 dpi (Fig. 3A). This suggests that there is no impairment in the induction of the antiviral response against RSV in HFD mice, a major deviation from the findings in IAV-infected HFD mice (12, 34). Proinflammatory cytokine concentrations were decreased in the lungs of HFD mice throughout infection; it is important to note, however, that these cytokine concentrations peaked at the same time in the lungs of SD and HFD mice, another important difference from IAV-infected HFD mice (9). The concentration of TNF-α was significantly lower in lung homogenates from HFD mice at 1, 4, and 7 dpi (Fig. 3B). IL-1β levels in the lungs of HFD mice were significantly lower at 1 dpi, but similar levels were detected in the lungs of SD and HFD mice at 4 and 7 dpi (Fig. 3B).

We also measured differences in immune mediators associated with T cell activation and differentiation. In lung homogenates, HFD mice had significantly reduced IFN-γ levels at 1 and 4 dpi compared to SD mice, although similar concentrations were detected at 7 dpi (Fig. 3C). At 4 dpi, IL-4 levels were below the lower limit of detection in HFD mice, while they were above detectable levels in SD mice (Fig. 3C). By 7 dpi, however, IL-4 levels were similar in the lungs of SD and HFD mice. Finally, levels of the immunomodulatory mediator IL-10 were significantly lower in lung homogenates from HFD mice at 7 dpi than in those from SD mice (Fig. 3C). Collectively, these results indicate differences in the lung microenvironments of SD and HFD mice, with HFD mice exhibiting modestly attenuated early proinflammatory and late immunomodulatory profiles during RSV infection.

### Innate immunity is altered in obese mice infected with RSV

**Fig 4:**
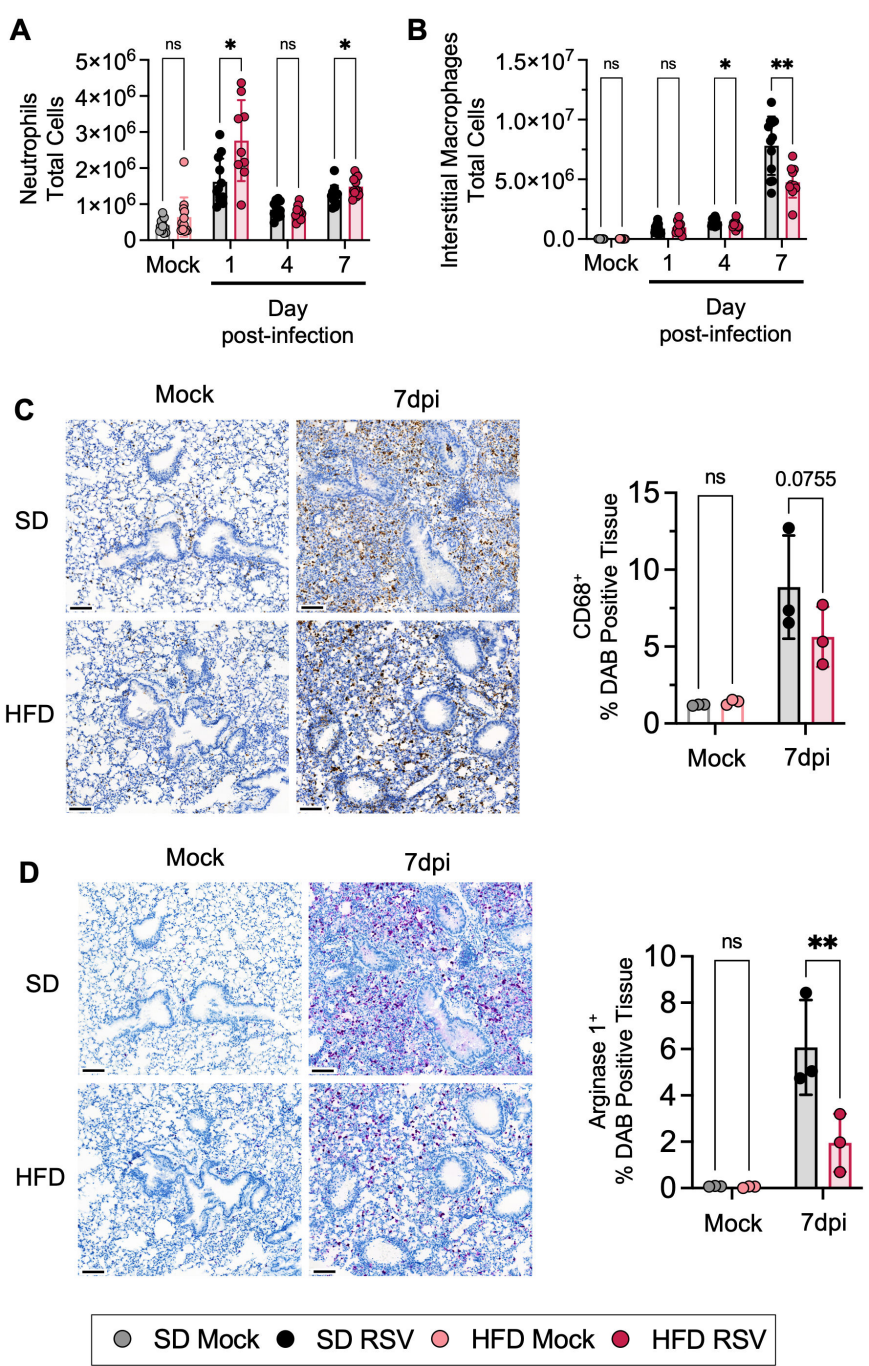
HFD mice have altered innate cell counts and immunomodulatory phenotype during RSV infection. Innate immune cell presence was determined by flow cytometry, and macrophage phenotype was determined by histological staining and IHC quantification. Total cell counts were determined for (**A**) neutrophils and (**B**) interstitial macrophages. *N* = 4 mice/diet group/experiment, three experiments. (**C**) Representative histological images of mock- and RSV-infected lungs from SD and HFD mice stained with CD68. Images were captured at 10×, and the scale bar equals 100 µm. Positive CD68 staining is in brown, and the negative counterstain is in blue. The graph represents IHC quantification for positive CD68 staining. *N* = 3 mice. (**D**) Representative histological images of mock- and RSV-infected lungs from SD and HFD mice stained with Arginase 1. Images were captured at 10×, and the scale bar equals 100 µm. Positive Arginase 1 staining is magenta, and negative counterstain is blue. The graph represents IHC quantification for positive Arginase 1 staining. *N* = 3 mice. Statistical significance for all parameters was determined using a mixed-effects model with Tukey-Kramer multiple comparisons. Asterisks represent *P* values for SD RSV compared to HFD RSV; *P < 0.05, **P < 0.01, ***P < 0.001, ns = not significant.

The above findings on antiviral cytokines indicate a decreased inflammatory profile in the lungs of HFD mice during early RSV infection. To determine whether this difference in inflammatory microenvironment is driven by, and subsequently impacts, lung immune cell profiles, we evaluated broad innate cell populations in the lungs of SD and HFD mice via flow cytometry at 1, 4, and 7 dpi. (Fig. S2) (35, 36). Unexpectedly, nearly twice as many neutrophils were detected in the lungs of infected HFD mice as in infected SD mice at 1 dpi (Fig. 4A). Neutrophil populations contracted by 7 dpi, with a slightly higher count in the lungs of HFD mice (Fig. 4A). HFD mice had significantly more inflammatory monocytes in their lungs prior to infection than SD mice (Fig. S3A). However, no differences were detected in monocyte counts between SD and HFD mice at 1, 4, or 7 dpi. HFD mice had significantly fewer macrophages in their lungs at 7 dpi (Fig. S3B). Although we did not observe any differences in alveolar macrophages at any of the tested time points (Fig. S3C), we detected a 2-fold decrease in interstitial macrophage counts in the lungs of HFD mice at 7 dpi (Fig. 4B). To determine if there was a difference in the macrophage phenotype between SD and HFD mice at 7 dpi, we stained paraffin-embedded lung tissue with CD68, a general marker for macrophages, and Arginase 1, a hallmark marker for M2 macrophages. CD68 staining trended lower in HFD mice at 7 dpi (*P* = 0.0755) (Fig. 4C), which aligns with our finding of reduced macrophage counts by flow cytometry (Fig. 4B; Fig. S3B). HFD mice had significantly reduced Arginase 1 staining at 7 dpi compared with SD mice (Fig. 4D). Overall, we observed modest differences in pulmonary innate immune cell populations between RSV-infected SD and HFD mice.

### CD8^+^ T cells are not impacted by diet during RSV infection

**Fig 5:**
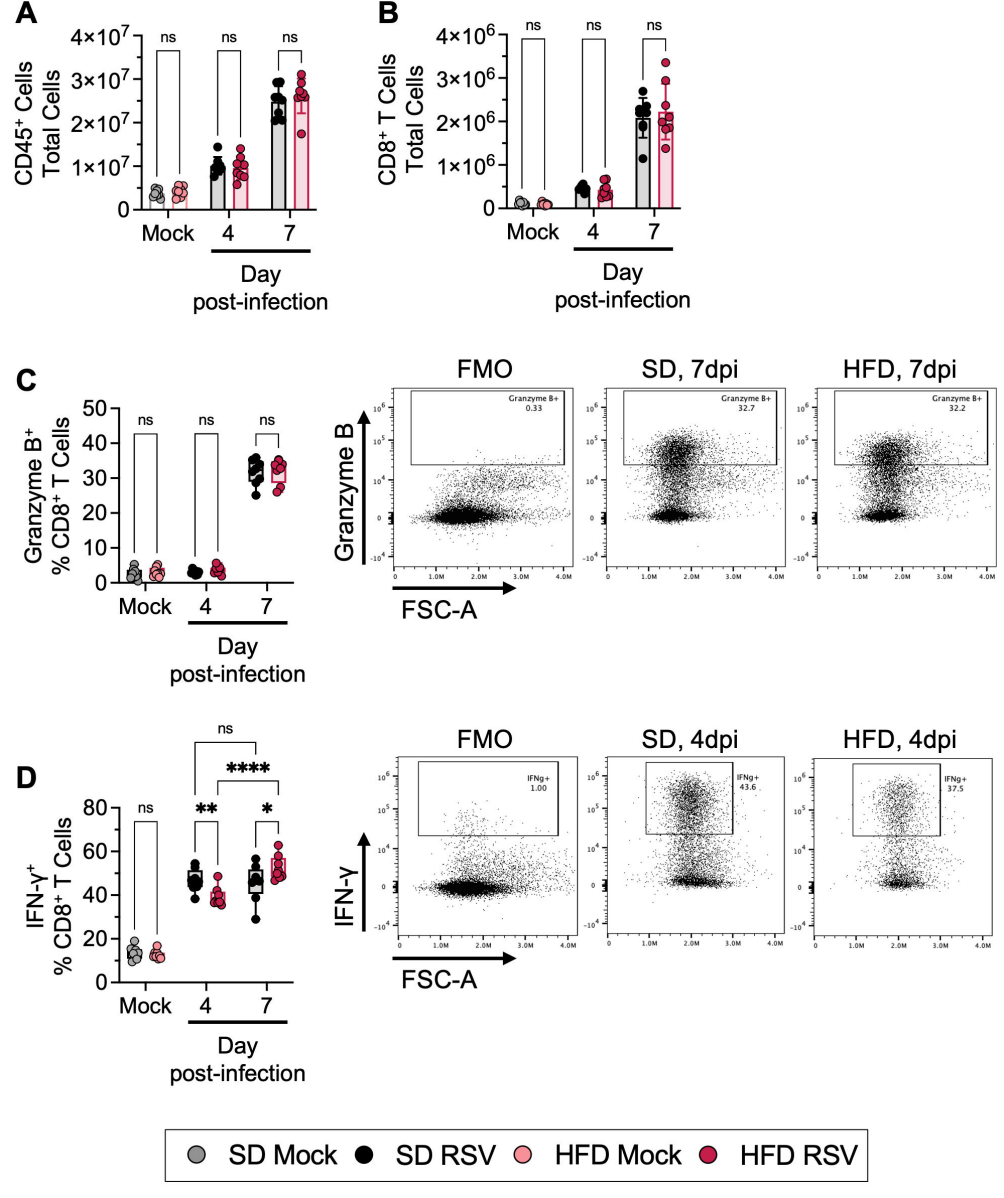
SD and HFD mice have comparable CD8^+^ T cell responses during RSV infection. Lungs from mock- or RSV-infected mice fed a SD or HFD were stimulated *in vitro* with PMA/Ionomycin in the presence of brefeldin A. Samples were subsequently stained for various extracellular and intracellular markers to determine CD8^+^ T cell counts and functionality. Total cell counts were determined for (**A**) live CD45^+^ cells and (**B**) CD8^+^ T cells. Total frequency was determined for (**C**) Granzyme B^+^ CD8^+^ T cells and (**D**) IFN-γ^+^ CD8^+^ T cells. *N* = 4 mice/diet group/experiment, two experiments. Statistical significance for all parameters was determined using a mixed-effects model with Tukey-Kramer multiple comparisons. Asterisks represent *P* values for SD RSV compared to HFD RSV; *P < 0.05, **P < 0.01, ****P < 0.0001, ns = not significant.

CD8^+^ T cells have been reported not only to be important for viral clearance but also to contribute to weight loss and increased lung parenchymal damage in mice infected with RSV (37, 38). Therefore, we sought to determine whether differences in CD8^+^ T cell responses between SD and HFD mice accounted for the observed differences in morbidity and histopathology (Fig. S4). We observed comparable counts of live CD45^+^ cells (Fig. 5A) and CD8^+^ T cells (Fig. 5B) between the two diet groups at 1, 4, and 7 dpi. To assess the differences in activation potential, we measured the frequencies of Granzyme B and IFN-γ-producing CD8^+^ T cells after stimulation with PMA and Ionomycin in the presence of brefeldin A. Diet did not affect the frequency of Granzyme B^+^ CD8^+^ T cells (Fig. 5C) in the lungs of mock- or RSV-infected mice at 4 or 7 dpi. At 4 dpi, the frequency of IFN-γ^+^ CD8^+^ T cells in the lungs of HFD mice was significantly lower than that of SD mice (Fig. 5D). While the frequency of these cells remained stable in SD mice between 4 and 7 dpi, expansion of this cell population was observed in HFD mice, resulting in a slight but significantly greater frequency of IFN-γ^+^ CD8^+^ T cells in the lungs of HFD mice at 7 dpi (Fig. 5D). The early proliferation of IFN-γ-producing CD8^+^ T cells in the lungs of SD mice at 4 dpi may contribute to the increased immunopathology observed in these mice (39).

### Diet impacts CD4^+^ T cell responses during RSV infection

**Fig 6:**
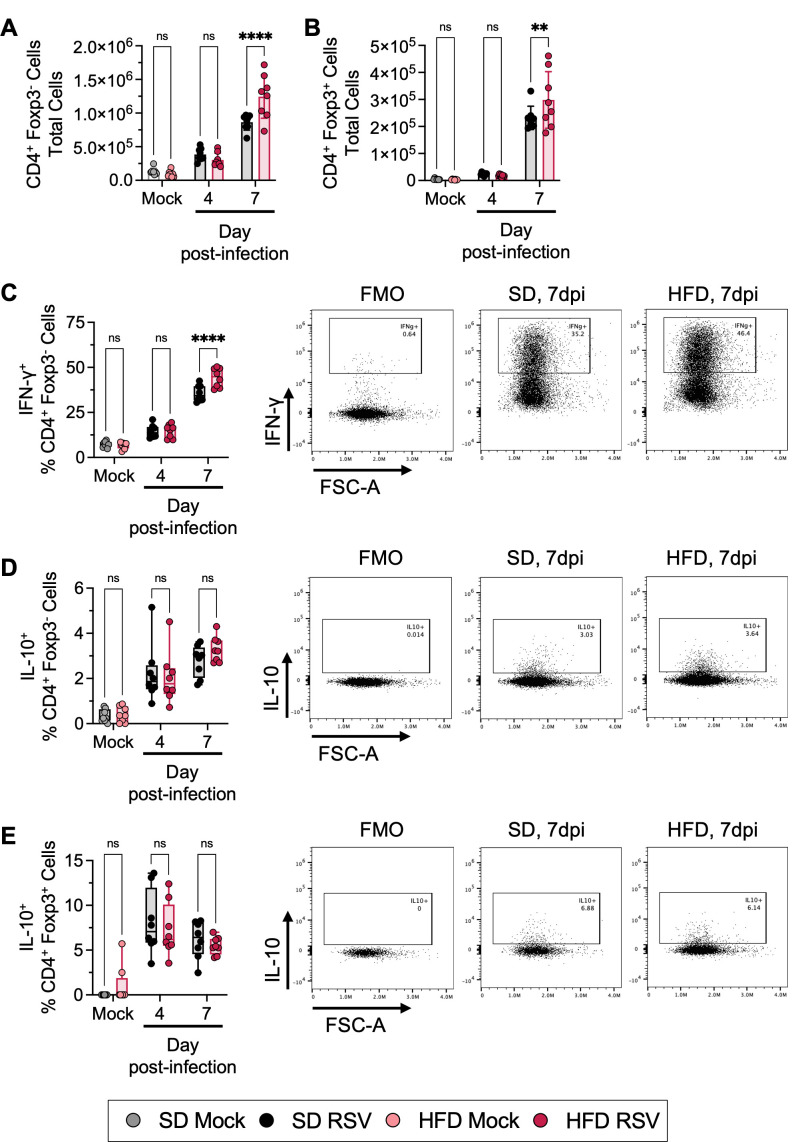
RSV-infected HFD mice have a more robust CD4^+^ T cell response than RSV-infected SD mice. Stimulated lungs isolated from mock- or RSV-infected SD and HFD mice were stained to determine CD4^+^ T cell counts and functionality. Total cell counts were determined for (**A**) CD4^+^ Foxp3^-^ T cells and (**B**) CD4^+^ Foxp3^+^ T cells. Total frequency was determined for (**C**) IFN-γ^+^ CD4^+^ Foxp3^-^ T cells, (**D**) IL-10^+^ CD4^+^ Foxp3^-^ T cells, and (**E**) IL-10^+^ CD4^+^ Foxp3^+^ T cells. *N* = 4 mice/diet group/experiment, two experiments. Statistical significance for all parameters was determined using a mixed-effects model with Tukey-Kramer multiple comparisons. Asterisks represent *P* values for SD RSV compared to HFD RSV; **P < 0.01, ****P < 0.0001, ns = not significant.

CD8^+^ T cells are essential for both resolving RSV infection and driving pathogenesis; however, CD4^+^ T cells are also important mediators in RSV disease (40). We observed differences in lung cytokine concentrations associated with T cell activation, namely IFN-γ and IL-10 (Fig. 3C). We therefore evaluated CD4^+^ T cell counts and activation potential at 4 and 7 dpi to determine their influence on disease progression in HFD mice (Fig. S2). At 7 dpi, HFD mice had significantly higher counts of CD4^+^ FoxP3^-^ T cells (Fig. 6A) and CD4^+^ FoxP3^+^ T cells (Fig. 6B) in their lungs compared with SD mice. After stimulation with PMA and Ionomycin in the presence of brefeldin A, we detected higher frequencies of IFN-γ^+^ CD4^+^ FoxP3^-^ T cells in the lungs of HFD mice at 7 dpi (Fig. 6C). Similar frequencies of IL10^+^ CD4^+^ FoxP3^-^ T cells (Fig. 6D) and IL10^+^ CD4^+^ FoxP3^+^ T cells (Fig. 6E) were detected in the lungs of SD and HFD mice at 4 and 7 dpi. Collectively, we observed an increase in CD4^+^ T cell populations in the lungs of RSV-infected HFD mice, accompanied by increased IFN-γ intracellular staining at 7 dpi.

## DISCUSSION

Obesity was identified as an independent risk factor for increased probability of hospitalization, mechanical ventilation, and death during the 2009 H1N1 influenza and the 2019 SARS-CoV-2 pandemics (5, 6). Investigative studies in animal models have shown that obesity is associated with prolonged viral replication, increased histopathology, and decreased survival (10, 13). These indicators of severe disease and poor outcomes in obese mice have been attributed to dysregulated antiviral immunity derived from low-grade systemic inflammation and intrinsically impaired immune cells (41, 42). Specifically, innate and adaptive immune cells from obese mice have been shown to exhibit reduced homing and infiltration capacity, as well as decreased activation potential, as determined primarily by cytokine production (43). Although the impacts of obesity on IAV disease progression have been well studied, relatively few other respiratory viruses have been thoroughly interrogated in the obese mouse model. In this study, we sought to characterize how obesity affects RSV pathogenesis in mice fed a HFD. We hypothesized that HFD mice would fare worse during RSV infection due to obesity-mediated dysregulation of antiviral immunity, as observed during IAV infections (3, 44). Contrary to this hypothesis, we found that HFD mice exhibited reduced weight loss and improved illness scores during peak RSV infection, less airway obstruction, and reduced alveolar-capillary permeability. These findings were associated with, and possibly explained by, lower viral loads and an altered antiviral and cellular immune response. It is likely that the mechanisms underlying the findings in this study are multifactorial and warrant future studies in animal models and in adults with obesity.

To begin untangling the driving forces behind the contrasting outcomes in IAV- and RSV-infected HFD mice, one must first consider the differences in disease manifestation between the two viruses. IAV is a rapidly replicating virus that releases viral progeny several hours after viral entry, and its highly cytopathic nature leads to extensive cell death and lung parenchymal damage (22, 34). Previous studies by Smith et al. and others have shown that airway immune cells in IAV-infected obese mice fail to secrete IFN-α and IFN-β, essential mediators for initiating antiviral immunity, leading to a delayed and pathological inflammatory immune response that synergizes with prolonged IAV replication and culminates in excessive histopathological damage and increased mortality in these obese mice (9, 10, 12, 45). An intriguing finding in the present study is reduced peak RSV loads in the lungs of HFD mice and comparable timing of viral clearance between SD and HFD mice, as determined through genome copies, plaque titers, and RSV N IHC staining (Fig. 2). RSV is a slower-replicating virus that does not release viral progeny until about 24–30 h after viral entry (46, 47). Importantly, RSV itself induces minimal cytopathology, with Zhang et al. demonstrating that primary cell cultures can persist for months in the presence of RSV (23). Instead, RSV indirectly influences disease severity by modulating the amplitude of the host immune response, with excessive or aberrant inflammation inducing immunopathology and driving disease severity (19, 32, 46). Previous studies evaluating the effects of RSV monoclonal antibodies in mice on lung viral titers and disease outcomes found that reduced viral loads were correlated with improvements in lung pathology and airway obstruction, as well as reduced cytokine production (33, 48, 49). These distinctive viral kinetics and drivers of disease manifestation in IAV and RSV infections may explain the differences in disease outcome in the HFD mouse model.

One key difference between the present study and IAV infection studies in obese hosts is in the induction of the antiviral response in RSV-infected HFD mice. The delayed and blunted type I IFN signaling in IAV-infected obese mice results in impaired antiviral responses and immune cell recruitment during the first few days of infection, leading to increased IAV spread and greater lung parenchymal damage (9, 10, 12). In contrast, we measured similar concentrations of IFN-α and IFN-β in the lungs of RSV-infected SD and HFD mice at 1 dpi (Fig. 3A). This finding suggests that the induction or primary source of type I IFNs may differ between IAV and RSV and that obesity does not negatively impact the initiation of the anti-RSV immune response. Although type I IFNs have been shown to be less essential for RSV control due to the antagonistic RSV proteins NS-1 and NS-2, these antiviral mediators are still necessary to induce a rapid protective response (50–52). Indeed, the trends of peak production for all evaluated cytokines were similar in RSV-infected SD and HFD mice (Fig. 3). The amplitude of the cytokine responses, however, differed between SD and HFD mice, with RSV-infected HFD mice having a more moderate inflammatory profile, which likely contributed to the reduced morbidity and histopathology observed at 4 and 7 dpi (Fig. 1D and E).

Innate immune cell counts measured throughout infection also indicate sufficient induction of the anti-RSV immune response in HFD mice. Diet did not impact inflammatory monocyte (Fig. S3A) or alveolar macrophage (Fig. S3C) counts in the lungs throughout RSV infection, although there were higher lung neutrophil counts in the lungs of HFD mice at 1 dpi (Fig. 4A). We also measured a significant difference in interstitial macrophages (IM) between HFD and SD mice at 7 dpi (Fig. 4B). IMs populate the lung parenchyma and mediate lung homeostasis and inflammation via the production of immunomodulatory mediators, such as IL-10 (53). We observed reduced IL-10 levels in the lungs of HFD mice at 7 dpi (Fig. 3C) that do not appear to be attributable to regulatory T cells (Fig. 6E) and instead may be due to differences in IM counts and phenotypes between SD and HFD mice. IL-10 induces an M2 macrophage phenotype, characterized by Arginase 1 expression, and these immunomodulatory mediators have been shown to correlate with increased trauma, presumably due to the induction of the wound-healing response (54–56). We observed that the lower IL-10 concentration coincided with reduced Arginase 1 expression in the lungs of HFD mice (Fig. 4D). This dampened M2 macrophage phenotype in the lungs of HFD mice is likely indicative of reduced histopathology rather than these cells contributing to lung damage in SD mice. The return of alveolar-capillary permeability to baseline levels (Fig. 1F) and histological resolution of disease (Fig. 1D) does not indicate impaired wound healing in RSV-infected HFD mice, as observed in IAV-infected HFD mice (10).

T cells have been identified as essential for controlling RSV replication, while concurrently being primary contributors to tissue damage, thereby driving disease exacerbation. Various studies have found that depletion of CD4^+^ or CD8^+^ T cells drastically impairs RSV clearance and mitigates lung damage (37, 57). In the present study, diet did not affect CD8^+^ T cell recruitment or activation potential (Fig. 5). This suggests CD8^+^ T cells are unlikely to be the primary drivers of viral control, morbidity, or immunopathology in the present study; however, future studies should evaluate the RSV-specific CD8^+^ T cell response in obese mice. At 7 dpi, HFD mice had increased counts of IFN-γ^+^ CD4^+^ Foxp3^-^ T cells in their lungs (Fig. 6), although no differences in IFN-γ concentrations were measured in the lungs of SD and HFD mice at this time point (Fig. 3C). CD4^+^ T cells generally exhibit a Th1 phenotype in obese hosts, and an increased proportion of IFN-γ^+^ CD4^+^ T cells has been observed in IAV-infected HFD mice (58, 59). This propensity for Th1 skewing in obesity could explain the increased frequency of IFN-γ^+^ CD4^+^ T cells detected in the lungs of HFD mice. Collectively, CD4^+^ and CD8^+^ T cells do not appear to play a critical role in the observed differences in disease severity between RSV-infected SD and HFD mice in this study.

In the present study, we found that mice fed a HFD exhibited reduced morbidity and histopathology upon intranasal infection with RSV A2. These findings were intriguing because obesity has previously been associated with worse disease progression and outcomes during IAV infections in both clinical and animal studies. These disparities in findings are likely due to the induction of a sufficient, albeit attenuated, antiviral response that coincides with a reduction in viral load in RSV-infected obese mice. It is possible that RSV’s slower replication cycle provides an opportunity for proper activation of the immune response in the obese host, resulting in adequate control and resolution of RSV infection. Reduced viral load, combined with intrinsic immune cell impairments in obesity, may result in a less robust inflammatory response, thereby mitigating disease severity. Future studies in animal models are needed to elucidate the mechanisms underlying improvements in disease progression during RSV infection, and more surveillance studies are needed to explore the individual risk of RSV-associated hospitalization in individuals with obesity.

There are limitations to our studies. RSV is only semi-permissive in mice and does not fully reflect disease presentation observed in humans. Although cotton rats are the preferred model for studying RSV viral kinetics, and BALB/c mice are the preferred model for studying RSV pathogenesis (60, 61), male C57BL/6 mice are the preferred model for studying the influence of immune dysregulation in diet-induced obesity (62), thereby making them the ideal animal model for this study. Many individuals with obesity have comorbidities that are well-defined risk factors for severe RSV disease, including cardiovascular disease and COPD. Therefore, our findings may not be broadly clinically translatable, and, importantly, we do not believe RSV vaccine regulations should be modified based on this study. However, the present study provides valuable insights into RSV pathogenesis and antiviral immunity in obesity. This study supports the idea that RSV disease severity is driven by excessive host immunity and highlights the roles of viral load and early innate inflammatory responses in subsequent disease severity. More importantly, our findings suggest that obesity does not unequivocally lead to worse disease across all respiratory viral infections; rather, it affects antiviral immunity and disease progression differently for individual respiratory pathogens. A better understanding of these nuances will help advance treatment and vaccine development for this high-risk population.

## SUPPLEMENTAL MATERIAL

Supplemental material mbio.00689-26-s0001.docx Supplemental figures and tables.

ASM does not own the copyrights to Supplemental Material that may be linked to, or accessed through, an article. The authors have granted ASM a non-exclusive, world-wide license to publish the Supplemental Material files. Please contact the corresponding author directly for reuse.
