# Supplementary material for: Mice on a high-fat diet have reduced immunopathology and an altered immune response during respiratory syncytial virus infection
Source: mBio. 2026 Apr 30;17(6):e00689-26. doi: 10.1128/mbio.00689-26 (PMC13251379; doi:10.1128/mbio.00689-26)
Supplement: Supplemental material — Supplemental figures and tables. [file mbio.00689-26-s0001.docx]

| 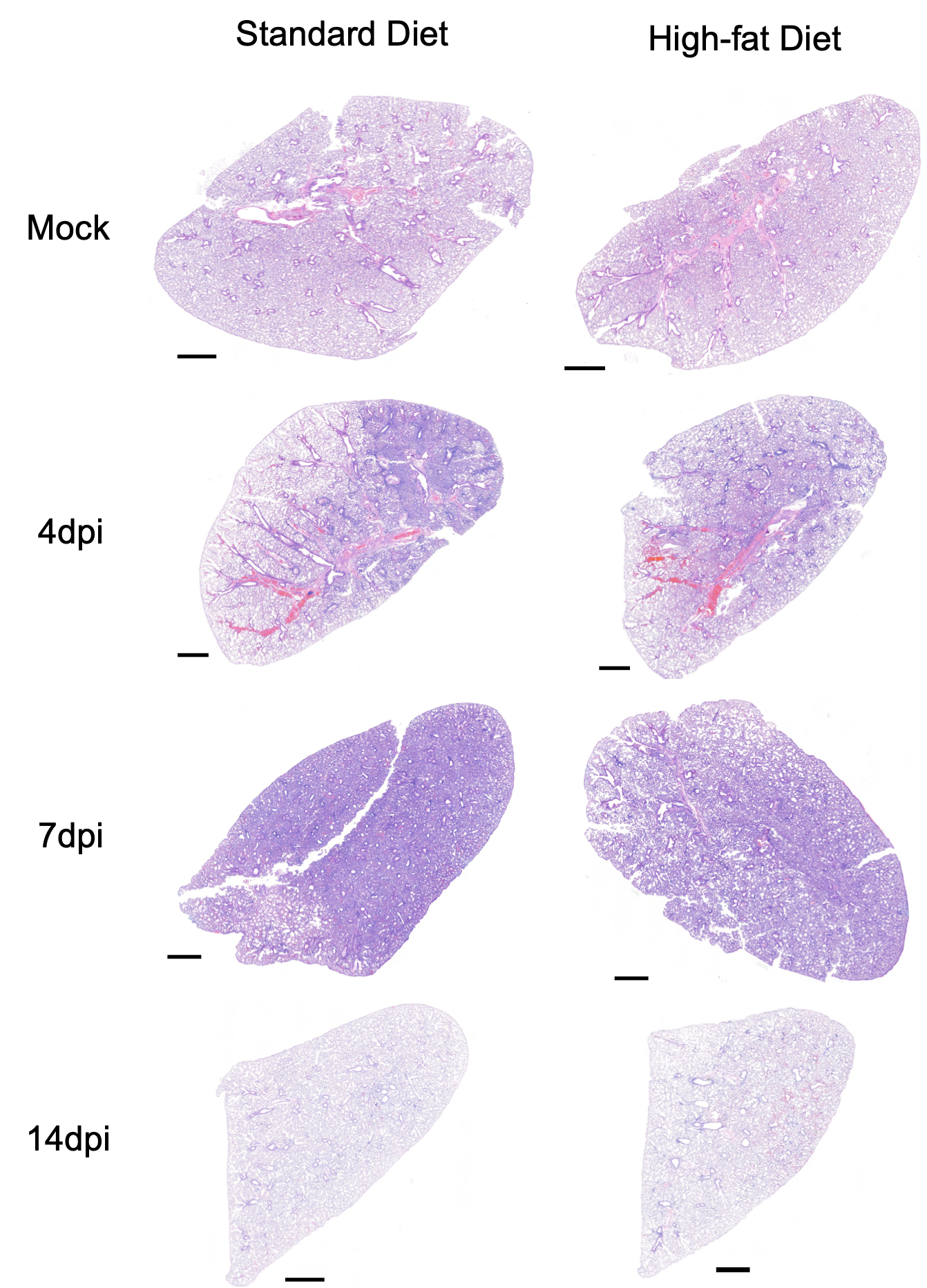 |
| --- |
| **Figure S1.** Representative histological slides of H&E-stained lungs harvested from mock- and RSV-infected SD and HFD mice. Matched whole lung images of the magnified lung images in **Fig 1D.** |

| 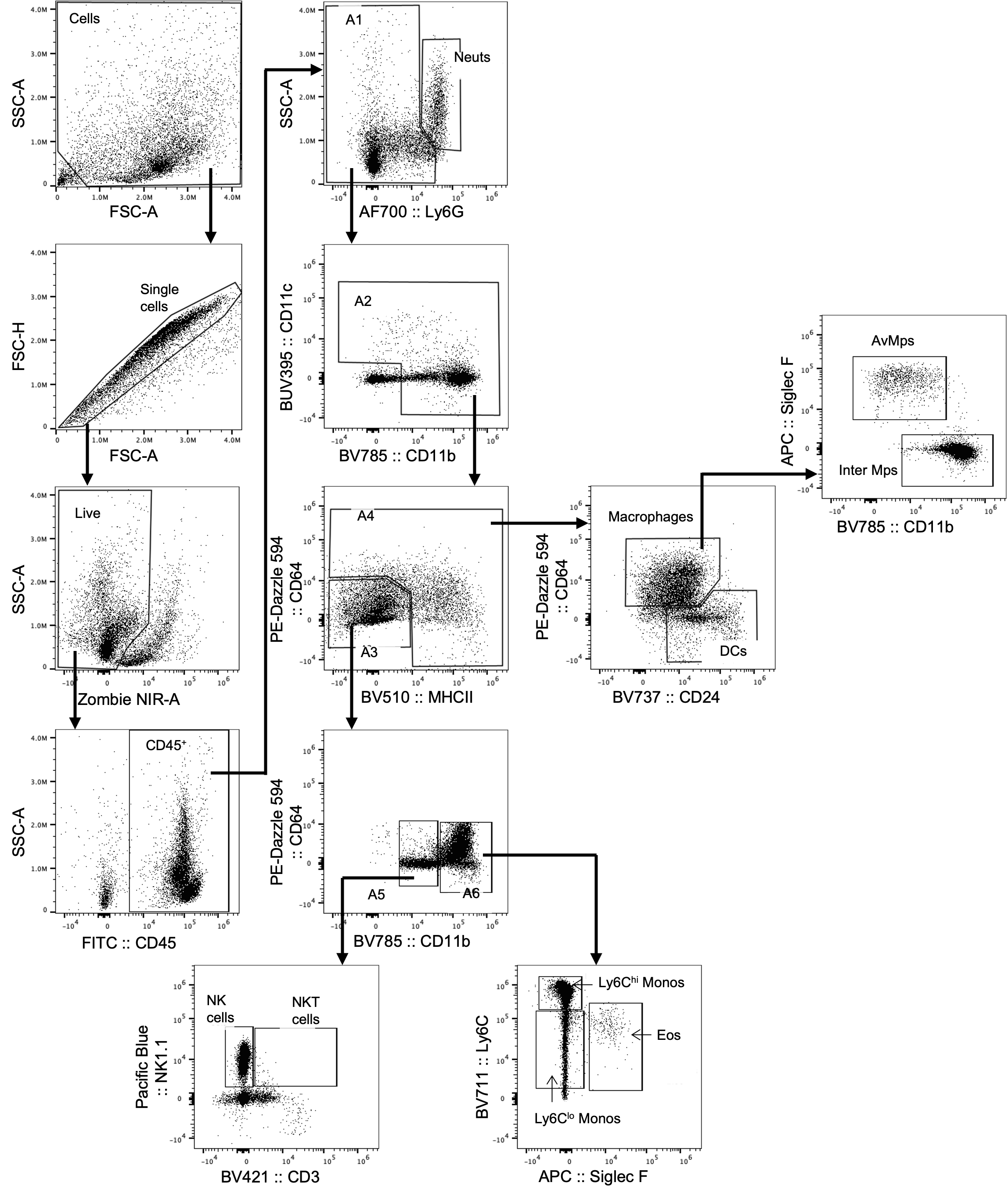 |
| --- |
| **Figure S2.** Gating strategy used for innate cells analyses shown in **Fig 4 and Fig S4.** |

| 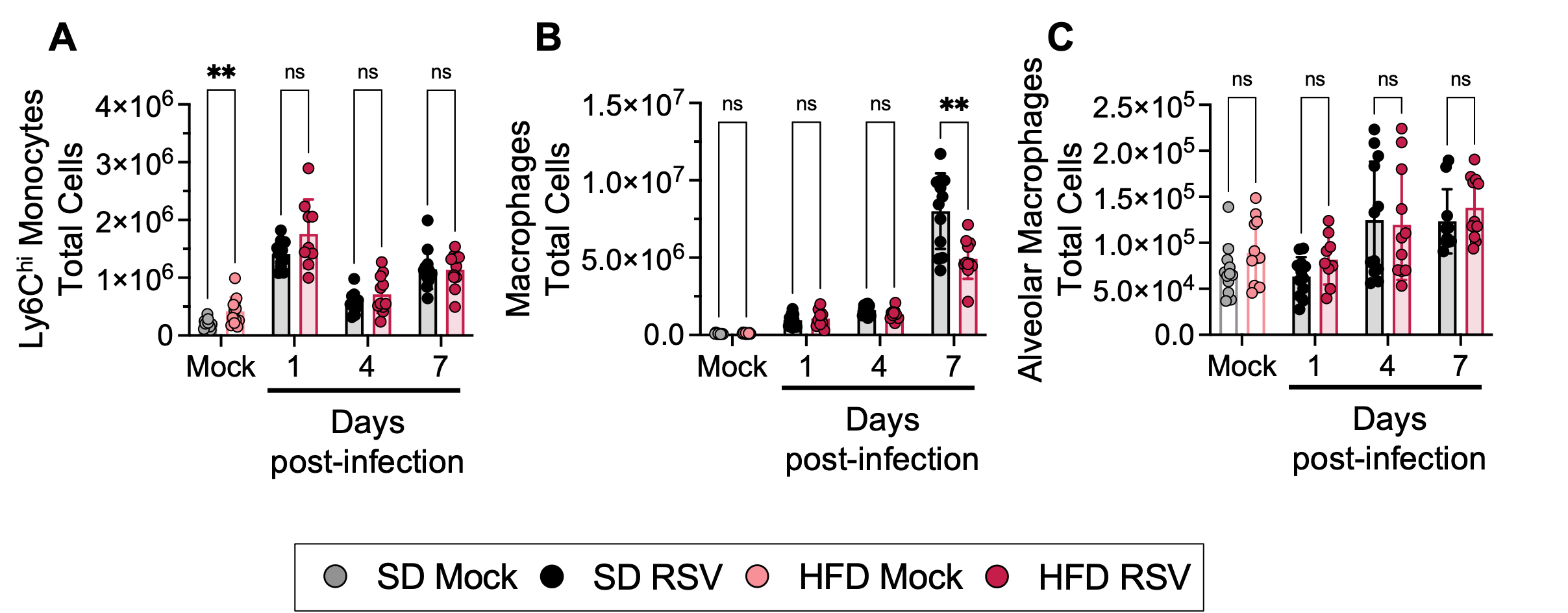 |
| --- |
| **Figure S3.** Total cell counts were determined for A) Ly6C^hi^ monocytes, B) macrophages, and C) alveolar macrophages. N=4 mice/diet group/experiment, 3 experiments. Statistical significance for all parameters was determined using a mixed-effects model with Tukey-Kramer multiple comparisons. Asterisks represent *p* values for SD RSV compared to HFD RSV; **<0.01, ns = not significant. |

| 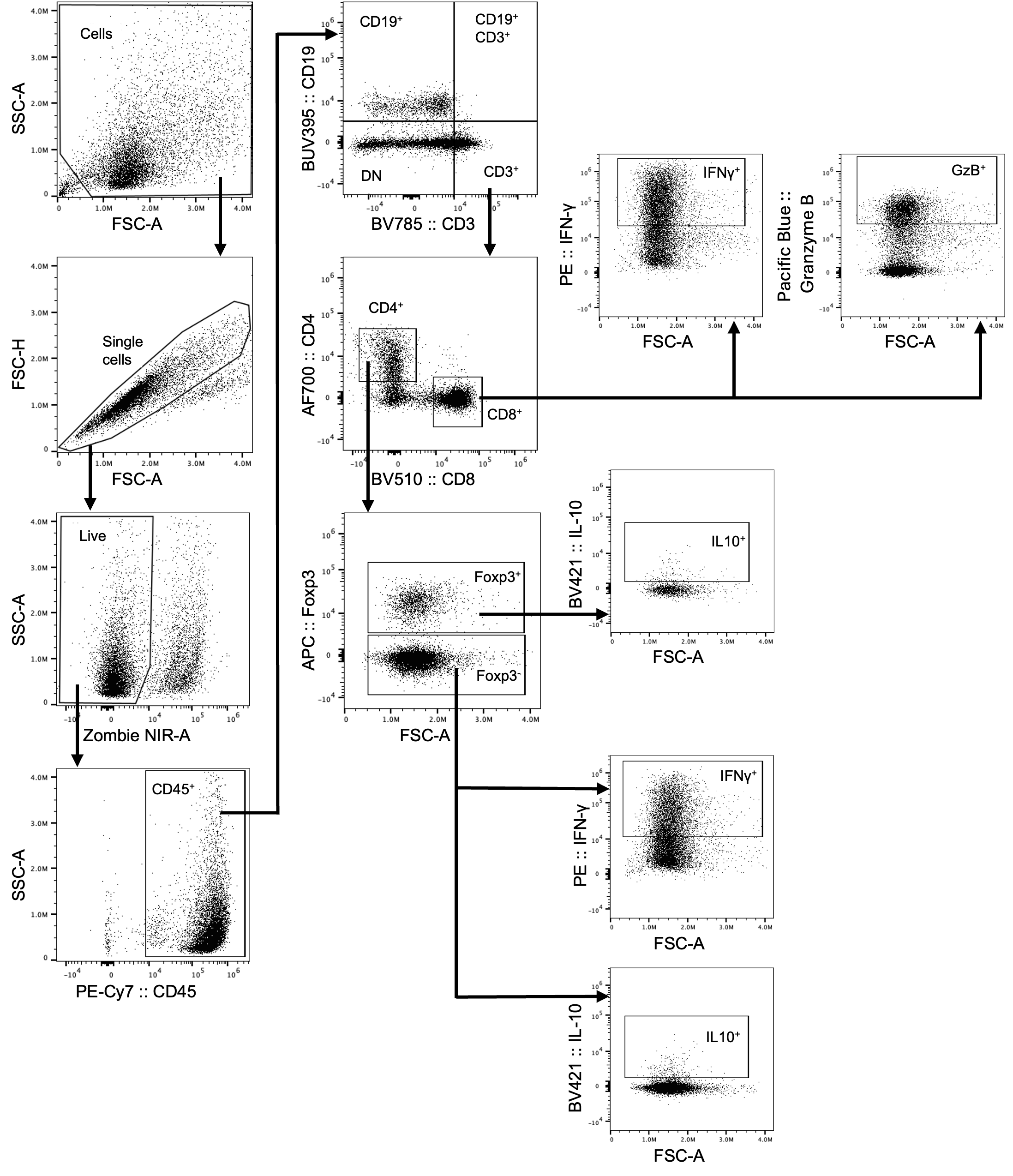 |
| --- |
| **Figure S4.** Gating strategy used for T cell analyses shown in **Figs 5** and **6**. |

**Table S1.** Antibodies for innate cell flow cytometry panel.

**Table S2.** Antibodies for T cell flow cytometry panel.
